# Psychiatric disorders and somatic comorbidities

**DOI:** 10.1192/j.eurpsy.2021.644

**Published:** 2021-08-13

**Authors:** A. Kerkeni, W. Abbes, Y. Mejdoub, M. Ben Hmida, S. Yaich, J. Dammak, L. Ghanmi

**Affiliations:** 1 The Department Of Psychiatry, Hospital of gabes, Gabes, Tunisia; 2 The Department Of Community Medecine, Hospital Hedi Chaker of Sfax, sfax, Tunisia; 3 The Department Of Community Medecine, Hospital Hedi Chaker, sfax, Tunisia

**Keywords:** Psychiatric disorders, Somatic comorbidities

## Abstract

**Introduction:**

People followed at the department of psychiatry have a high prevalence of somatic pathologies that are generally not taken optimal care of in time, which implies excess mortality rate among these patients.

**Objectives:**

To study somatic comorbidities in patients followed at the department of psychiatry of the regional hospital of Gabes (Tunisia).

**Methods:**

We conducted a retrospective, descriptive and analytical study carried out on a clinical population who consult for the first time at the psychiatry department at the Gabes regional hospital during the period from January 1st, 2010 to December 31, 2013. Sociodemographic, clinical and therapeutic data of the patients were assessed. Data were analysed using the software SPSS (20th edition).

**Results:**

The number of patients consulting for the first time at the psychiatry department during the study’s period was 1601 patients, with a mean age of 34 years and a sex ratio (M / F) of 0.96. Among these patients, 399 (24.9%) had somatic comorbidity. The most common somatic comorbidity was arterial hypertension (8.1% of patients, n=129 patients). Diabetes mellitus was ranked second with 99 patients (6.2%). The analytical study showed that depressive disorders were significantly more frequent in patients with hypertension (p<0.001), diabetes mellitus (p<0.001) and asthma (p=0.026).

**Conclusions:**

Somatic comorbidities were frequent in patients followed by the department of psychiatry. Paying attention to somatic comorbidities must be part of the evaluation of these patients in order to coordinate effectively with the somatic doctors.

